# Fractal Profit Landscape of the Stock Market

**DOI:** 10.1371/journal.pone.0033960

**Published:** 2012-04-27

**Authors:** Andreas Grönlund, Il Gu Yi, Beom Jun Kim

**Affiliations:** 1 Department of Mathematics, Uppsala University, Uppsala, Sweden; 2 BK21 Physics Research Division and Department of Physics, Sungkyunkwan University, Suwon, Korea; Philipps-University Marburg, Germany

## Abstract

We investigate the structure of the profit landscape obtained from the most basic, fluctuation based, trading strategy applied for the daily stock price data. The strategy is parameterized by only two variables, *p* and *q* Stocks are sold and bought if the log return is bigger than *p* and less than *–q*, respectively. Repetition of this simple strategy for a long time gives the profit defined in the underlying two-dimensional parameter space of *p* and *q*. It is revealed that the local maxima in the profit landscape are spread in the form of a fractal structure. The fractal structure implies that successful strategies are not localized to any region of the profit landscape and are neither spaced evenly throughout the profit landscape, which makes the optimization notoriously hard and hypersensitive for partial or limited information. The concrete implication of this property is demonstrated by showing that optimization of one stock for future values or other stocks renders worse profit than a strategy that ignores fluctuations, i.e., a long-term buy-and-hold strategy.

## Introduction

Everyone wants to be rich, who doesn’t? As a way of investment, stock market provides not only a chance to become a millionaire, but also a direct shortcut to pennilessness. In the present paper we focus on two important properties of the stock trading industry. First, it is shown that individual households pay a tremendous performance penalty for active trading [1]. Even professional fund managers cannot outperform the market indices in long term [2]. Second, many stock traders can be characterized as chartists, in principle using stock charts solely to make trading decisions [3], [4]. Inspired by the above two observations, we will try to make some conclusions regarding the chances of actually beating the market trend by exploiting temporal variations in the stock market. We will not try to perform a complete modeling of interacting traders, nor will we model all possible trading strategies, but rather learn from a very simple strategy that exploits the fluctuations of stock prices and see whether it can shed any light to the observed difficulties in beating the market trend.

We admit that the trading strategies used in reality could be much more complicated and sophisticated than the naive strategy in this work. Various technical trading strategies based on moving averages, price momentum, channel breaking, and relative stock index have been being used [5]–[7]. In technical trading, suitably defined signals of buying and selling are produced from the past stock price movements, and trading strategies can typically be grouped into two different categories: trend-following and contrarian. The stock trading strategy in this work is parameterized by only two parameters that will be used for quantifying how fluctuations propagate to the long-term profit of the strategy. It should be noted that we are not aiming at proposing a profitable trading strategy, but we hope to understand the structure of the profit landscape yielded from a very basic trading strategy of buying and selling. However, by composing a very simple strategy from elementary buying and selling signals, more complex strategies can be composed from combinations of such signals, and robust features of our strategy, revealed by the profit landscape, should hold also for such, more complex, strategies.

## Methods

In this work, we use 95 US company stocks (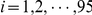), which existed for 21 years between 1983 and 2004. The stock price 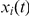 is given for the *i* -th stock at time 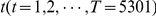, which is the consecutive integer increasing by one at every trading date. What we mean by a strategy *S* in this paper is the way of determining (i) whether or not to trade (buy or sell), and if it is decided to trade (ii) how many units of stocks are to be traded. Only for simplicity, we assume that if ‘buy’ decision is made in (i), we buy number of stocks by spending the *f_b_* fraction of cash. Likewise, if ‘sell’ decision is made, *f_s_* fraction of stocks in possession are sold. Accordingly, our imaginary simple portfolio (we trade stocks for only one company, i.e., *i* fixed) is composed of only two accounts; cash *m*(*t*) and number *n*(*t*) of stocks, and the estimated value of the portfolio is given as the sum of the cash and stocks, 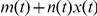. In reality, the amount of cash alone can increase in time by trading the risk-free asset, which is not taken into account in the present study only for the sake of simplicity. In other words, we assume that the risk-free interest rate is zero. In the same spirit, we also neglect the increase of cash via dividends provided by companies. We note that our expression for the value of the portfolio is the same as that of the wealth in Ref. [8]. In order to quantify a strategy with a small set of parameters, we propose the following simple strategy (we call it *S*_1_):

- The trade decision at time *t* depends only on the stock prices at two distinct times *t* and 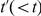. More specifically, the log return 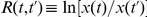 is used for the trade decision.
- If 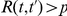, one judges that the stock price went up much and thus decides to sell.
- If 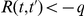, the decreased stock price makes the stock attractive to buy, and thus one decides to buy.

We also consider the inverse strategy called *S*_2_:

- If 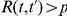, one expects that the stock price will go up later and decides to buy.
- If 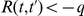, one is afraid of further price falling, and thus decides to sell.

In words, the strategy *S*_1_ can be termed as “sell-on-rise/buy-on-fall”, whereas the inverse strategy *S*_2_ as “sell-on-fall/buy-on-rise”. Similarly, the trend-opposing (i.e., contrarian) and the trend-following strategies have been used in Ref. [9], although the meaningfulness of the concept of “trend” in real markets is questionable [2]. For the sake of simplicity, we impose the non-negative cash constraint (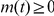) [9] and the non-negative stock constraint (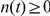). In other words, you can neither buy stocks if you do not have enough cash, nor you can sell stocks which you do not have (short selling is not allowed). We also assume that the stock price is given exogenously and that the transaction price of the stock trading equals the daily closure price in the data file.

**Figure 1:**
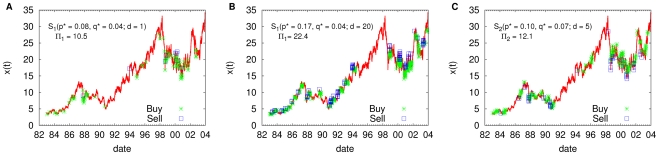
Price time series and strategy-dependent trading decisions. The time series of the stock price for a company is plotted as a function of time (full line), and the time instants when buy and sell decisions are made for given strategies are marked as star and square symbols, respectively. For the resolution 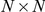 (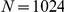) in the parameter space of 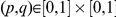, we compute the profit 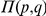 at 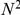 points to find the global profit maximum at 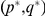. The used strategies are (A) 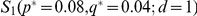, (B) 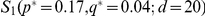, and (C) 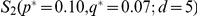. For all cases, we used 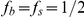. Depending on the strategy used, the instants when buy and sell decisions are made are very different from each other, and the resulting profit values are also quite different: 10.5, 22.4, and 12.1 in (A), (B), and (C), respectively.

In general, the strategy in the present setup can be written as 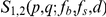 with 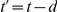 (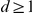). For given values of 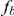, 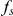, and *d*, we investigate the performance of the investment strategy parameterized by the two variables *p* and *q*. The detailed algorithm of performing our investment game is as follows: (i) We pick a company *i* one by one. (ii) At the first trading date, we start from one million dollars 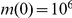. (iii) For given values of *p* and *q*, we keep applying the strategy repeatedly till the last date *T*. (iv) We evaluate the performance of strategy by computing the profit defined by 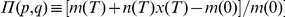. For each stock trading, we also assume that we pay small trading fee 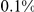. In the real stock market, there often exits minimum number of stocks to be traded. We have compared 1, 10, and 100 as minimum trading volume, only to find insignificant change of results. Henceforth, our strategies allow the trade of a single unit of stocks (but you cannot sell or buy a fraction of a stock).

**Table 1:** Strategy set. For *S*_1,2_, the buy/sell decisions are made according to the log-returns of the stock price with the time difference *d*.

strategy	description
*S*_0_	buy-and-hold
*S*_1_	sell-on-rise and buy-on-fall
*S*_2_	buy-on-rise and sell-on-fall

## Results

We first compute the profit 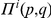 of historic stock data for the *i*-th company, in the two-dimensional parameter space 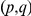 defined on unit square 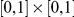. For convenience, we discretize the parameter space into an 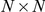 square grid and compute *N* ^2^ values of 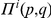 at the center of each small square of the size 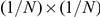. Once those values are computed, one can easily find the global maximum of the profit 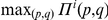 for the given resolution *N*, and call those optimal values as 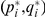. We also calculate the number *M* of local maxima which satisfy the criterion that four neighboring points in square grid have smaller values of the profit than at the center. As an example, we run the simulations at 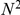 (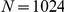) grid points for 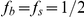 and then use the obtained optimal value 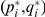 to construct Fig. 1, where we also denote time instants when the buy and the sell decisions are made.

**Figure 2:**
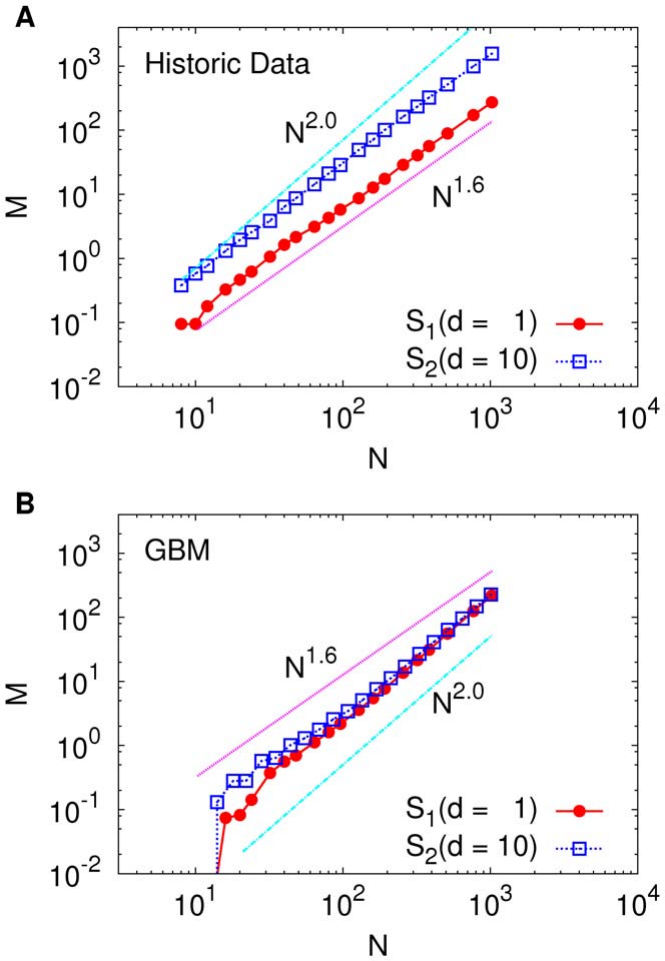
Scaling of the number of local maxima in the profit landscape. (A) The number *M* of local maxima of the profit landscape [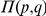] is computed as a function of the number *N* of grid points in the two-dimensional parameter space. The local maxima of the profit are distributed like a geometric fractal, manifested by 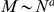 with 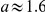. For comparison, we also display the curve for 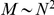, which clearly deviates from the actual result. (B) *M* versus *N* calculated from the GBM time series (see text). For GBM, 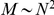 fits better to the result than 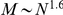.

Whether or not short term fluctuations in the stock market may be exploited is addressed by benchmarking our strategies with a “buy-and-hold” strategy *S*_0_ (see Table 1): At 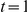 all cash is spent to buy stocks and the profit is evaluated at 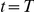, given by 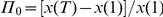. The value of 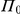 changes from company to company, depending on the growth rate of each firm. The growth rate conditioned to the firm size has been known to have exponential distribution function [10], while the size of each firm is power-law distributed [11]. For a company shown in Fig. 1, 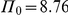, which is smaller than the maximum profit realizable by the strategies *S*_1_ and *S*_2_. The actual profit of any strategy for historic data is however of marginal interest; more interesting issue to pursue is what we can learn from the profit landscape and see if it allows us to make money in the future.

We next study how the number *M* of local maxima of the profit landscape defined on the *p*-*q* plane changes as the resolution parameter *N* is increased. If the profit landscape has a simple structure that there are only a few number of peaks, *M* is expected to first increase with *N*, since the number of peaks found in the higher resolution (larger *N*) will be larger than that in the lower resolution (smaller *N*). In this case of a simple landscape, *M* will then soon saturate to an 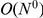 value, and will not increase any more even when *N* is increased further. If this is indeed the case, the optimization process we described above can be very efficient to locate the global maximum profit. If the maxima are uniformly distributed they will scale linearly with the number of boxes 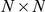, that is 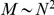. In Fig. 2A we show how *M* (averaged over 95 USA stocks) changes as a function of *N* for different strategies. The scaling falls between the two extremes: Surprisingly, *M* increases with *N* following an algebraic form of 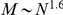 for both strategies. Although not reported here, we find that the behavior 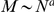 with 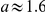 for broad range of parameter values of 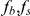, and *d*, both for *S*_1_ and *S*_2_. The same value of *a* is found when extended range 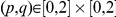 is used, and when eight neighbors of square grid (instead of four nearest neighbors) are compared for the determination of local maxima. Furthermore, Korean stock price data also reveal the same behavior with 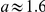 (to be reported elsewhere). These observations clearly indicate that the pattern of how local maxima are scattered in the two-dimensional parameter space is described by a *fractal* structure.

**Figure 3:**
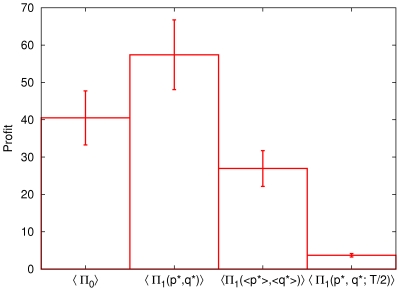
Performances of different strategies. 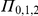
 is the profit from the strategy 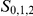 (see Table 1), and 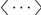 is the average over all companies. See text for details.

We next compare our findings to the standard model of stock price, i.e., geometric Brownian motion (GBM) [12]:
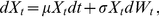
(1)where 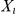 is the Brownian random variable and 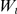 is the Wiener process. The parameters 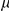 and 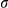 are fitted to each of the stocks and a number of replicas are simulated over the same period of the real stock data. A striking difference to the real stock price data is that for the GBM local maxima are evenly distributed, seen by the scaling 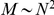 in Fig. 2B. A conclusion we can make from this is that the profit cannot be explained solely from the first and the second order statistical moments (the mean value and variance) but a more detailed description of stock price movement is needed. It is well known that GBM is not able to describe some stylized facts of real price movements such as high value of kurtosis, fat tails in the probability distributions of log-returns, and the stochastic volatility and its clustering behavior [13]–[17]. Beyond GBM various stochastic processes have been proposed with Lévy processes as the most prominent example. Various models as subclasses of Lévy processes and autoregressive models have been proposed [13]–[15], [18]. The results from more sophisticated models of financial-time series are however not further corroborated here.

**Figure 4:**
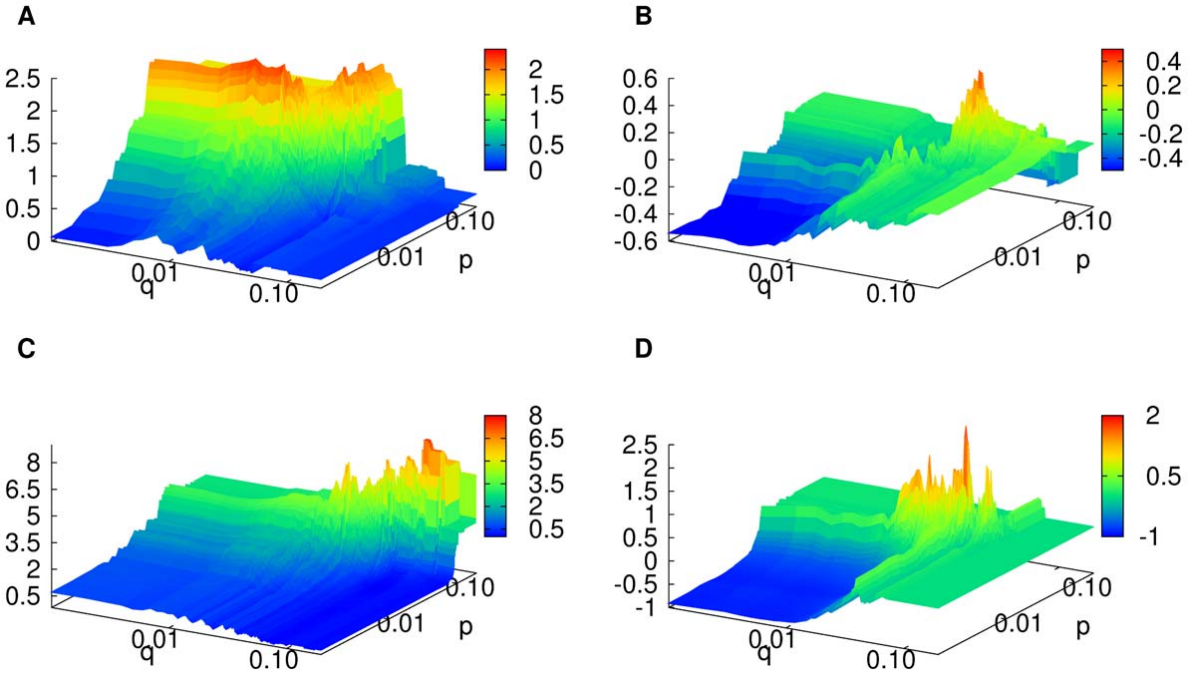
Profit landscape in two-dimensional parameter space. (A) 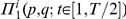 and (B) 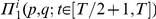 are for the same stock (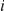) but for different time periods, and (C) 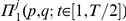 is for other stock (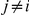). The three landscapes (A)–(C) look different, in agreement with the instability of optimal value of 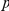 and 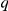 over different stocks and different time periods. (D) 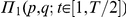 for a GBM time series. For better visibility in the small values of *p* and *q*, we plot the landscapes in the plane of *p* and *q* in log scales. For (A)–(D), the strategy *S*_1_ is used.

Although all the strategies belonging to *S*_1_ and *S*_2_ have qualitatively the same fractal structure in their profit landscapes, the maximum profit realizable by a given strategy is very different from each other. In general, the average maximum profits of *S*_1_ are found to be larger than those of *S*_2_. This can also be seen in Fig. 1B and C: In *S*_1_ sell prices are often higher than the buy price, while the opposite is seen for *S*_2_. Similarly, contrarian strategies have been shown to yield higher profits than trend-following ones in [5].

We also observe that for given values of 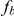 and 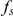, the average maximum profit does not significantly depend on the value of *d*. This is not a surprising observation since it is well known that the autocorrelation of returns decays very fast (in several minutes) [19], and thus the time difference *d* larger than one day will not make much difference in the results.

We have seen that there exist fluctuation based strategies for which the maximum profits significantly exceed the profit from the long term buy-and-hold strategy 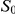. The question is how to find the optimal strategy, since we need to estimate 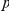 and 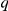 for future stock data by using past stock data. Even if we possibly cannot find the *optimal* strategy by optimizing for old data we would like to know whether we can be sure to find a *good* strategy from such an optimization process. The fractal profit landscape suggests that the optimization process is very sensitive to perturbations, such as missing data and the change of optimization period of time.

We study the stability of the optimized strategies in two different ways: First, we compare how the profit changes across different companies for given values of *p* and *q*. More specifically, we obtain the optimized values 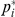 and 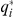 for each company 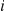, and use the average values 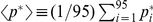 and 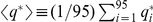 to compute the profit by the strategy 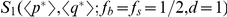 applied for each company 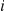, which gives us the average profit 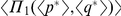. Second, we compare how the profit changes in different time periods as follows: We use the first half period (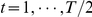) for optimization of the strategy, and then use the obtained values 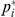 and 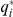 to compute the profit for the second half period (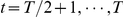). These two ways to test the stability of the optimized strategy can be phrased as the tests for spatial stability (across stocks 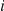) and for temporal stability (across the time *t*).

Figure 3 summarizes the results from the stability tests of the optimized strategy. It is again shown that the optimized strategy for each individual company for the whole period yields better profit than that from the buy-and-hold strategy, i.e., 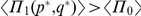. Since each stock has different time evolution behavior, the optimized values 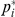 and 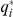 are different for different companies. In this regard, it makes some sense to use 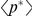 and 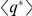 for all the companies, expecting that these values could have somehow better performance, although not as good as 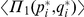. The third box denoted as 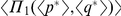 in Fig. 3 clearly shows that the use of the average values 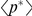 and 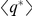 does not give us better profit than the buy-and-hold strategy. In Fig. 3, we also display the result from the temporal stability test, denoted as 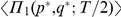. It shows that the use of the optimized strategy for later time periods dramatically reduces the profit value. The same conclusion is reached when we use finer time windows as follows: We divide the whole time period *T* into 20 time intervals and obtain the optimized value 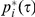 and 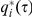 at each 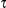-th interval (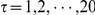). We then use 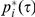 and 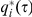 to compute 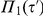 at later time interval 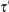 (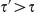). Even the largest value of the profit [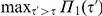] is found to be smaller than the profit from the buy-and-hold strategy at the same interval. This indicates that our conclusion of the temporal instability of the optimal strategy holds at least for sufficiently long time scales.

The results from the stability tests can be summarized as follows: Even though fluctuations, theoretically, may be exploited in our basic trading scheme, the 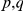-parameters (i) cannot be estimated from historic data and (ii) nor can they be estimated from other stocks. In both cases the performance is significantly worse than the buy-and-hold strategy. Our results agree with other existing studies: It is now generally believed that stock market has become more efficient and most technical trading strategies based on daily price changes stopped being profitable in mature market [5], although developing markets can still be different [7].

We next investigate the actual shapes of the profit landscapes in terms of the observations made above. Figure 4 displays (A) 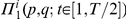 and (B) 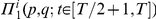 for one stock (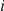), and (C) 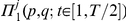 for other stock (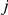). One can see that the shape of the landscape looks quite different from each other, supporting the above conclusion of the instability of optimal strategy. It is also seen that the profit landscape is quite rugged in accordance with the fractal-like distribution of the local maxima reported in the present study. For comparison, we also show in Fig. 4D the profit landscape for the GBM series. The difference of the fractal dimension for local maxima between real stock prices and GBM is not clearly seen in Fig. 4, however, the landscape for (D) GBM looks more rugged than the landscape for (A) an actual stock.

## Discussion

In summary, we have investigated the profit landscape defined by a set of simple investment strategies. We have shown that the local maxima in the profit landscape are scattered like a fractal and that the global profit maximum increases very slowly as the search resolution in the parameter space is increased. These findings imply that a local search in a strategy space to get the highest profit is almost impossible. We believe that our conclusions of poor performances of fluctuation-based trading strategies and their fractal profit landscapes are related with the unavoidable lack of future information of a company. If one has full information of the stock price change for the future, that information can directly be used to optimize trading strategy. However, if the future information is not sufficiently accurate, it can be basically useless in increasing profit, as has already been shown in Ref. [20]. We plan to study the rugged profit landscape in the present work in comparison with the fitness landscapes in other research areas [21].
